# Multimodal deep learning model integrating electronic medical records and CT images for gallbladder cancer diagnosis: a retrospective multicenter study in China

**DOI:** 10.1186/s12885-026-16116-w

**Published:** 2026-05-11

**Authors:** Ziming Yin, Yijun Shu, Dacong Shen, Hongyu Gao, Yijue Zhang, Ziyi Yang, Wei Gong, Tao Chen, Yingbin Liu

**Affiliations:** 1 School of Health Science and Engineering, University of Shanghai for Science and Technology, Shanghai, 200093 China; 2 Department of General Surgery, Xinhua Hospital, Shanghai Jiaotong University School of Medicine, Shanghai, 200092 China; 3 Department of Biliary and Pancreatic Surgery, Renji Hospital, Shanghai Jiaotong University School of Medicine, Shanghai, 200127 China; 4 Shanghai Key Laboratory of Systems Regulation and Clinical Translation for Cancer, Shanghai, 200127 China; 5 State Key Laboratory of Systems Medicine for Cancer, Shanghai Cancer Institute, Shanghai, 200127 China; 6 Department of Anesthesiology, Renji Hospital, Shanghai Jiaotong University School of Medicine, Shanghai, 200127 China

**Keywords:** Gallbladder cancer, Adaptive weighting, Multimodality, Radiomics

## Abstract

**Objective:**

Gallbladder cancer (GBC) is a rare gastrointestinal malignancy with a global 5-year survival rate of less than 5%. Early diagnosis is challenging owing to the lack of specific clinical symptoms. Additionally, the high heterogeneity of gallbladder tumors limits the clinical utility of unimodal deep-learning methods for GBC diagnosis. This study aimed to develop a novel multimodal deep-learning model to facilitate the preoperative diagnosis of GBC in more patients.

**Methods:**

We conducted a retrospective multicenter study using contrast-enhanced arterial phase computed tomography (CT) images and laboratory examination data from 300 patients (150 GBC cases and 150 non-GBC cases) extracted from electronic medical records of two Grade A tertiary hospitals in Shanghai between 2018 and 2020. A novel two-stage multimodal diagnostic model (GBC-DiagNet) was developed: the first stage achieved coarse segmentation of the gallbladder region using a position-constrained 3D Attention U-Net (improved by combined sampling) to avoid over-segmentation; the second stage realized GBC detection via an adaptive feature fusion strategy, which optimizes the weighted integration of handcrafted radiomic, deep radiomic and laboratory examination features to enhance diagnostic performance.

**Results:**

On the independent test set, the model achieved an accuracy of 0.933 (95% confidence interval [95% CI]: 0.927–0.94), specificity of 0.912 (95% CI: 0.904–0.922), sensitivity of 0.962 (95% CI: 0.937–0.986), precision of 0.893 (95% CI: 0.875–0.911), an F1-score of 0.926 (95% CI: 0.919–0.932) and AUC (area under the curve) of 0.9706 (95% CI: 0.961–0.981). Compared with the optimal unimodal model, our model improved accuracy, sensitivity, and F1-score by 14.28%, 16.76%, and 16.85%, respectively. Furthermore, compared to state-of-the-art deep-learning architectures (ResNet, DenseNet, MobileNet, ConvNeXt, ViT), our model exhibited absolute improvements of 7.68% in accuracy, 8.03% in F1-score, and 0.0059 in AUC.

**Conclusion:**

The proposed multimodal model integrating contrast-enhanced CT and laboratory data achieves stable and clinically meaningful diagnostic performance for gallbladder cancer, supporting its utility as an artificial intelligence-assisted tool for preoperative noninvasive diagnosis.

## Background

Gallbladder cancer (GBC) is a malignancy of the biliary system and ranks sixth among gastrointestinal cancer types worldwide. In recent years, the global incidence of GBC has risen, with China reporting the highest number of cases [1]. Most GBC patients are asymptomatic in the early stages, leading to delayed diagnosis. Unfortunately, GBC cells readily metastasize through the blood and lymphatic systems, often making surgical intervention impractical at the time of diagnosis. Currently, the median survival time for GBC patients is only six months, and the 5-year survival rate is merely 5% [2]. Timely diagnosis and treatment of GBC are thus crucial for improving the prognosis of GBC patients. In the early stages, GBC is easily misdiagnosed with benign gallbladder diseases, such as gallbladder polyps, adenomyosis, xanthogranuloma, and gallstones. Accordingly, clinicians and researchers have sought to develop artificial intelligence (AI)-based methods to assist in GBC diagnosis.

In clinical practice, a comprehensive evaluation of multiple clinical features is required to assess the likelihood of GBC. Thus, integrating multiple data sources—rather than relying on a single source—is essential when applying AI techniques for GBC diagnosis. Deep learning technology can fuse heterogeneous multimodal data to extract complementary diagnostic information (e.g., tumor markers and visual imaging cues). Fusion strategies are generally categorized as early, middle, and late fusion [3–6]. Early fusion directly combines multimodal data for feature extraction [7]. Middle fusion involves feature-level fusion after extracting features from each individual modality [8, 9]. Late fusion performs fusion at the decision-making level [10, 11]. Early fusion is suitable for fusing homogeneous multimodal data, whereas middle and late fusion are more appropriate for heterogeneous multimodal data [12–14]. Imaging examinations (contrast-enhanced computed tomography [CT]) and laboratory tests (tumor marker assays) are fundamental for GBC diagnosis. We therefore adopted a middle fusion strategy—specifically a feature-level fusion method—for heterogeneous multimodal data. This approach effectively correlates radiomic features with laboratory test data features and enables the model to learn the correlational information between these feature sets.

In clinical GBC diagnosis, clinicians typically extract shallow image features using handcrafted radiomic processes, which involve close examination of regions of interest (ROIs) based on expert knowledge. However, gallbladders are small organs with significant morphological variability, and cancerous regions can present with a wide range of shapes [15], making them extremely difficult to identify with the naked eye. Human expert detection often overlooks deep image features that could improve diagnostic accuracy [16]. Deep-learning-based radiomic techniques [17, 18] overcome this limitation by applying multiple continuous neural convolutions [19, 20]. These techniques integrate low-level and high-level features for comprehensive organ segmentation [21, 22], leveraging the complementarity of these features [23]. For these reasons, fusing handcrafted radiomic features and deep-learning-based radiomic features is necessary to characterize the complete image features of GBC.

The primary aim of this study was to develop a multimodal deep learning diagnostic model (GBC-DiagNet) for GBC by integrating laboratory examination data and contrast-enhanced CT images. The model employs a two-stage multimodal fusion network to combine laboratory data, handcrafted radiomic features, and deep radiomic features. First, contrast-enhanced CT images undergo coarse segmentation to localize potential gallbladder regions (including normal, benign, and cancerous tissues), with the improved 3D Attention U-Net to mitigate the model’s over-segmentation. Second, multimodal features are fused via targeted feature extraction methods and input into the diagnostic model. Compared with previous models, these innovations preserve more valuable feature information and enhance the accuracy of GBC diagnostic model.

## Methods

### Overview

**Fig. 1:**
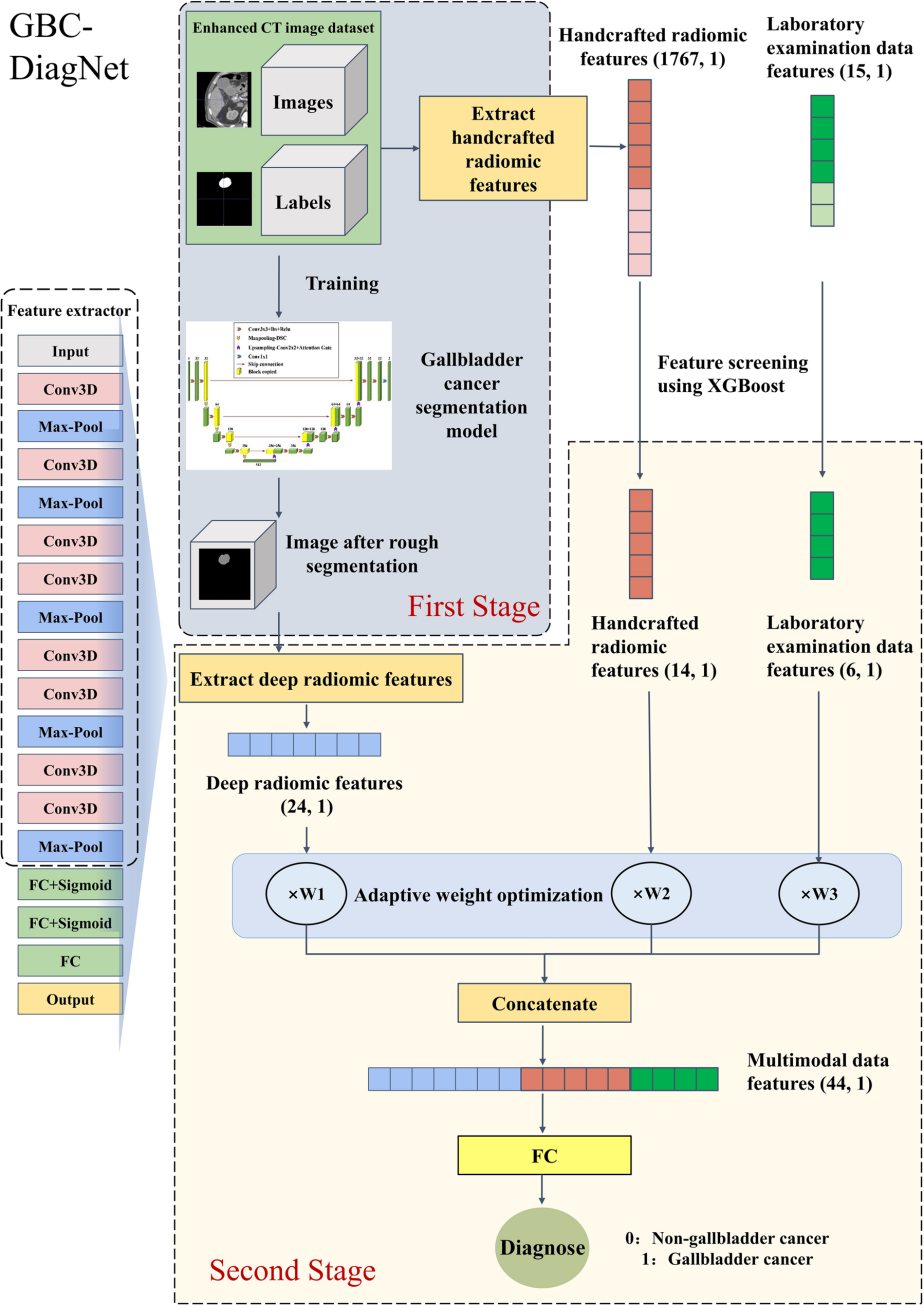
Structure of GBC diagnostic model based on adaptive weighted multimodal feature fusion

The overall architecture of GBC-DiagNet is illustrated in Fig. 1. The model consists of two core stages: coarse segmentation of the gallbladder region and multimodal data feature modeling with adaptive weighting. First, the XGBoost algorithm [24] is used to screen laboratory examination data features and handcrafted radiomic features based on their importance scores. The coarse segmentation results of the gallbladder region are then input into a custom-designed 3D convolutional neural network (3D-CNN). The 3D convolution outputs (256 × 3 × 2 × 1) are flattened into a one-dimensional vector (1,536 × 1), which is further reduced to deep radiomic features (24 × 1) using two fully connected (FC) layers. Finally, the deep radiomic features (24 × 1), handcrafted radiomic features (14 × 1), and laboratory examination data features (6 × 1) are weighted and concatenated into a single multimodal data vector (44 × 1). This vector is input into a classifier for GBC prediction.

### Patients and heterogeneous multimodal dataset

We retrospectively collected data from eligible patients at two large Grade A tertiary hospitals in Shanghai between 2018 and 2020. Patients with missing imaging or laboratory data were excluded from the study. Additional exclusions were made due to imaging-related issues identified during physician annotation, including missing arterial-phase CT images or low-quality CT scans. The remaining 300 patients constituted the study cohort, including 150 patients with pathologically confirmed GBC—pathological examination is the gold standard for cancer diagnosis. The other 150 non-cancer patients included 62 cases of gallbladder polyps, 7 cases of xanthogranuloma, 43 cases of adenomyosis, and 38 cases with normal gallbladders.

#### Contrast-enhanced CT image dataset

Contrast-enhanced CT images are acquired by injecting a contrast agent into the target organ to highlight tissue differences between normal and diseased states. These images are typically categorized into arterial phase, portal venous phase, and delayed phase. Arterial phase images enable clinicians to identify potential lesions more effectively, so our dataset was constructed using carefully selected arterial phase contrast-enhanced CT images. All images were in DICOM format with a resolution of 512 × 512. The window width was 300, and the window level was 30. Two board-certified radiologists with 8 and 12 years of abdominal imaging experience independently annotated the primary GBC lesions on these images. Lesion boundaries were defined based on arterial phase enhancement characteristics and pathological reports. Discrepancies between the two annotators were resolved through consultation with a third radiologist with 15 years of clinical experience. The inter-annotator agreement was quantified using Cohen’s Kappa coefficient, yielding a Kappa value of 0.82 indicating excellent inter-rater consistency. All annotations were performed using 3D Slicer (version 4.11), and the annotated ROIs were exported in NIfTI format for subsequent model training.

#### Demographic and laboratory examination dataset

Demographic data included patient gender and age. Among the 150 GBC patients, 64 were male and 86 were female, resulting in a male-to-female ratio of 1:1.34. Among the 150 non-cancer patients, 82 were male and 68 were female, with a male-to-female ratio of 1.2:1. Tumor markers (e.g., CA19-9 and carcinoembryonic antigen) provide an important baseline for cancer diagnosis. The complete distribution of demographic and laboratory examination data is presented in Table 1.

**Table 1:** Demographic and laboratory examination data of patients

Characteristics	GBC (*n* = 150)	Without GBC (*n* = 150)	*P* value
Gender			0.024
Male	64 (42.7%)	82 (54.7%)	
Female	86 (57.3%)	68 (45.3%)	
Age (year) (median [IQR])	66 [59.25, 73.75]	62 [53.0, 68.0]	< 0.001
PT (s) (median [IQR])	11.2 [10.6, 12.0]	11.5 [11.1, 12.2]	< 0.001
INR (10^− 1^) (median [IQR])	1.0 [0.95, 1.08]	1.05 [1.00 1.11]	< 0.001
APTT (s) (median [IQR])	29.8 [27.1, 32.3]	31.7 [29.4, 34.1]	< 0.001
FIB (g/L) (median [IQR])	3.62 [2.92, 4.26]	2.96 [2.56, 3.40]	< 0.001
TT (s) (median [IQR])	16.0 [13.8, 17.9]	14.7 [13.8, 15.5]	< 0.001
WBC (10^9^/L) (median [IQR])	5.94 [4.9, 7.55]	5.7 [4.44, 7.2]	0.08
Hb (10^1^/L) (median [IQR])	123 [110, 130]	130 [119, 140]	0.023
PLT (10^9^/L) (median [IQR])	220 [176, 274]	196 [157, 237]	0.02
AFP (ng/mL) (median [IQR])	2.775 [1.96, 4.22]	2.49 [1.87, 3.79]	0.24
CEA (ng/mL) (median [IQR])	3.04 [1.85, 6.97]	2.40 [1.42, 3.95]	< 0.001
CA19-9 (U/mL) (median [IQR])	74.05 [15.04, 204.5]	11.9 [7.21, 23.0]	< 0.001
CA125 (U/mL) (median [IQR])	19.8 [11.58, 48.47]	11.7 [8.72, 15.1]	< 0.001
CA72-4 (U/mL) (median [IQR])	2.45 [1.50, 5.95]	1.84 [1.50, 3.85]	0.25
T stage			-
T1	19 (12.7%)		
T2	32 (21.3%)		
T3	72 (48.0%)		
T4	10 (6.7%)		
Tx	17 (11.3%)		
N stage			-
N0	96 (64.0%)		
N1	35 (23.3%)		
N2	7 (4.6%)		
Nx	12 (8.0%)		
M stage			-
M0	128 (85.3%)		
M1	14 (9.3%)		
Mx	8 (5.3%)		
Stage			-
0	6 (4.0%)		
I	18 (12.0%)		
II	23 (15.3%)		
III	83 (55.3%)		
IV	20 (13.3%)		

*PT *Prothrombin time,* INR *International normalized ratio,* APTT *Activated partial thromboplastin time,* FIB *Fibrinogen,* TT *Thrombin time,* WBC *White blood cell,* Hb *Hemoglobin,* PLT *Platelet,* AFP *Alpha-fetoprotein,* CEA *Carcino-embryonic antigen,* CA19-9 *Carbohydrate antigen 199,* CA125 *Cancer antigen 125,* CA72-4 *Carbohydrate antigen 724

Based on the normal reference ranges, each indicator value was categorized into two discrete classes. Specifically, continuous values were transformed into binary features representing two states (coded as 1 for normal, 0 for abnormal). This discretization simplifies anomaly handling and enhances model generalization. Additionally, gender was encoded using one-hot encoding. For other indicators including patient age, routine blood test results (WBC, Hb, PLT), and coagulation function parameters (PT, INR, APTT, FIB, TT), min-max normalization was applied to scale values to the range [0, 1]. However, it should be noted that the discretization of continuous laboratory indicators may result in partial loss of detailed data information. In addition, the classification based on fixed normal reference ranges has a certain degree of subjectivity, which may affect the feature distribution and consequently impose a potential impact on model training and predictive results.

### Handcrafted radiomic feature extraction

The PyRadiomics (v.3.0.1) package in Python (v.3.7.0) was used to extract radiomic features from 3D images using a variety of filtering techniques. The filters included Laplacian of Gaussian, Wavelet, Square, Square root, Logarithm, Exponential and Gradient. The extraction parameters were set as follows: Bin width = 25, Log kernel size = 1, 2, 3, 4, 5, and Resampled voxel size = 3, 3, 3. A total of 1,767 radiomic features were extracted, including 342 first-order statistics, 456 Gy-level co-occurrence matrix, 304 Gy-level size zone matrix, 304 Gy-level run length matrix, 266 for gray-level dependence matrix and 95 neighboring gray tone difference matrix features.

**Fig. 2:**
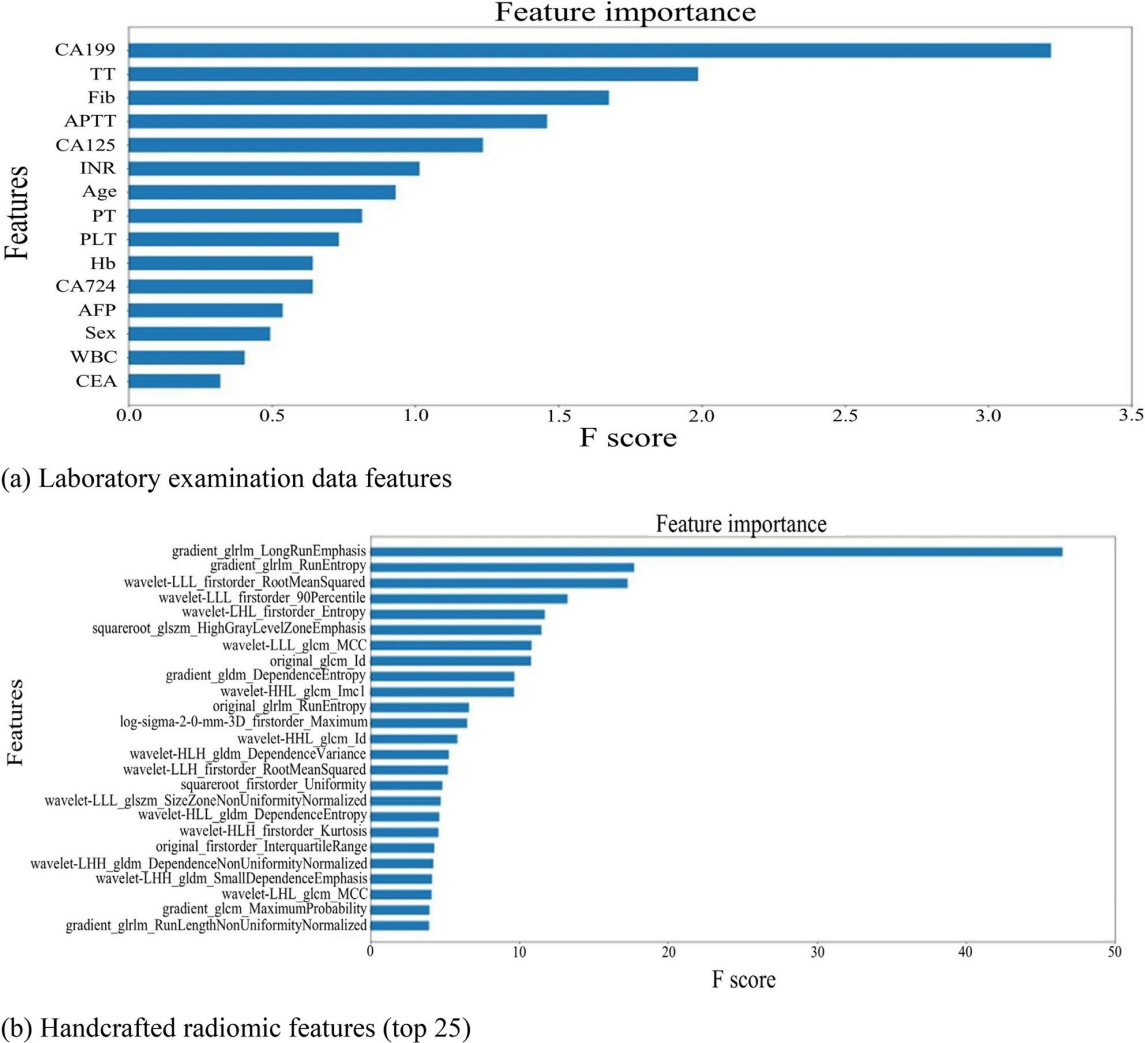
Importance rankings of two kinds of features. **a** Laboratory examination data features, (**b**) Handcrafted radiomic features, (top 25)

We used the XGBoost feature screening algorithm to calculate the importance scores of 15 types of laboratory examination data features and 1,767 handcrafted radiomic features (Fig. 2). Only the top 25 features are displayed in the figure. Based on the changes in relative importance score values, we selected the top 6 laboratory examination data features and the top 14 handcrafted radiomic features for subsequent model training.

### Position-constrained 3D attention U-net

We proposed a position-constrained 3D Attention U-Net model based on reconstruction pooling for GBC segmentation. This model modifies the traditional 3D Attention U-Net by replacing its single pooling layer with a combined structure of a pooling layer and a convolutional layer. This modification addresses two key limitations of conventional pooling layers: significant semantic information loss and the absence of learnable parameters. Furthermore, the segmentation results from the reconstruction-pooling-based 3D Attention U-Net were post-processed using a position-constrained network. This step effectively mitigates image over-segmentation, a common issue in medical image segmentation tasks.

#### Position-constraint network

**Fig. 3:**
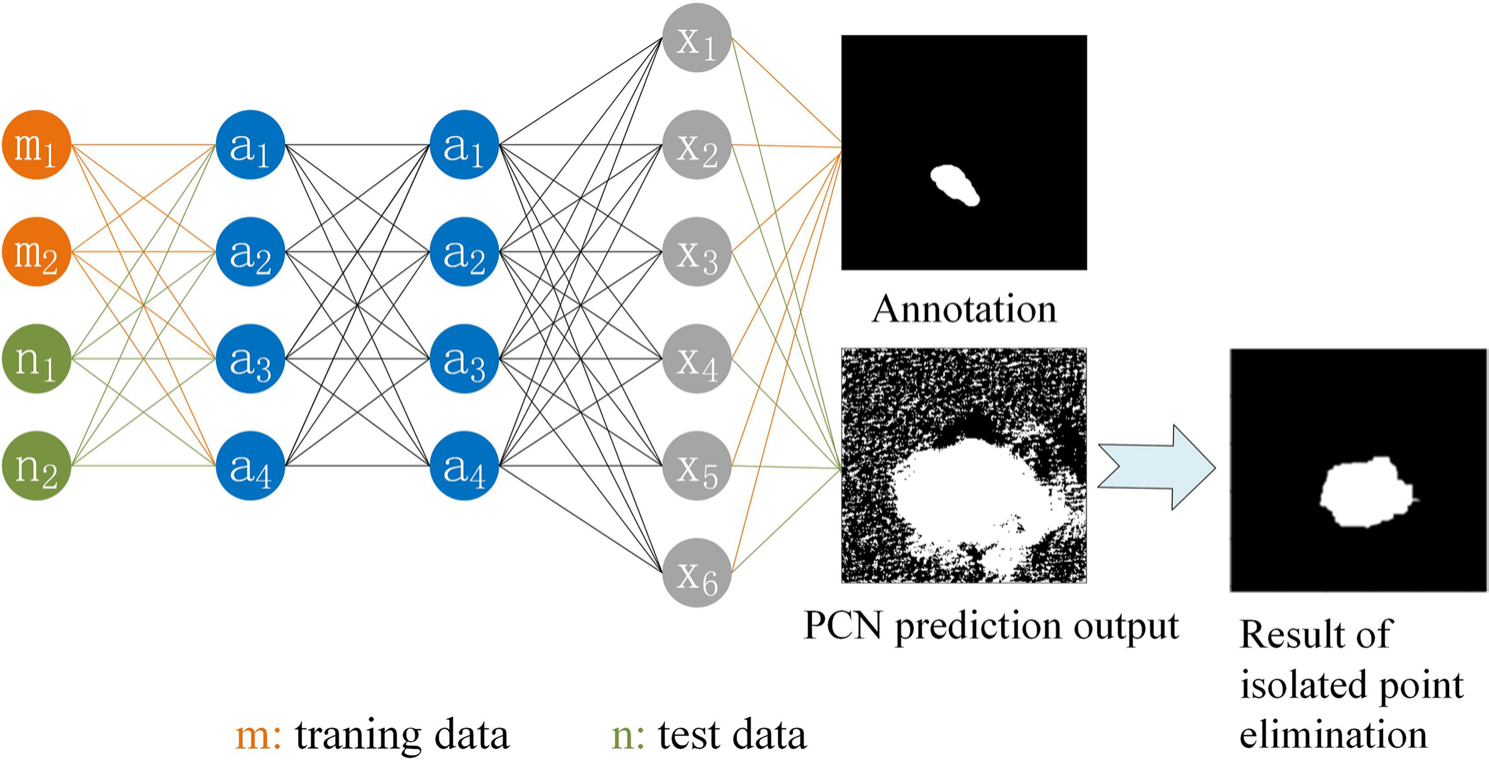
PCN structure

The Position-Constraint Network (PCN) is a deconvolution layer based on a learning network, which is the inverse operation of a convolutional layer. Convolutions streamline and extract image information for classification tasks, while deconvolutions map extracted features back to the original target image space. Our PCN deconvolution operation discards inconspicuous features while maintaining a clear correspondence between retained features and the target image. The structure of the PCN is illustrated in Fig. 3.

#### Combined sampling

Traditional pooling layers aggregate pixel values via downsampling of input feature maps, which often causes positional information loss and reduced segmentation accuracy. To enhance model performance, we replaced the downsampling MaxPooling layer of the conventional 3D Attention U-Net with a new “MaxPooling-DSC” unit. This unit combines traditional MaxPooling with depthwise separable convolution (DSC). Similarly, the standard upsampling layer was replaced by an “UpSampling-Conv” unit, which integrates upsampling and convolution operations.

**Fig. 4:**
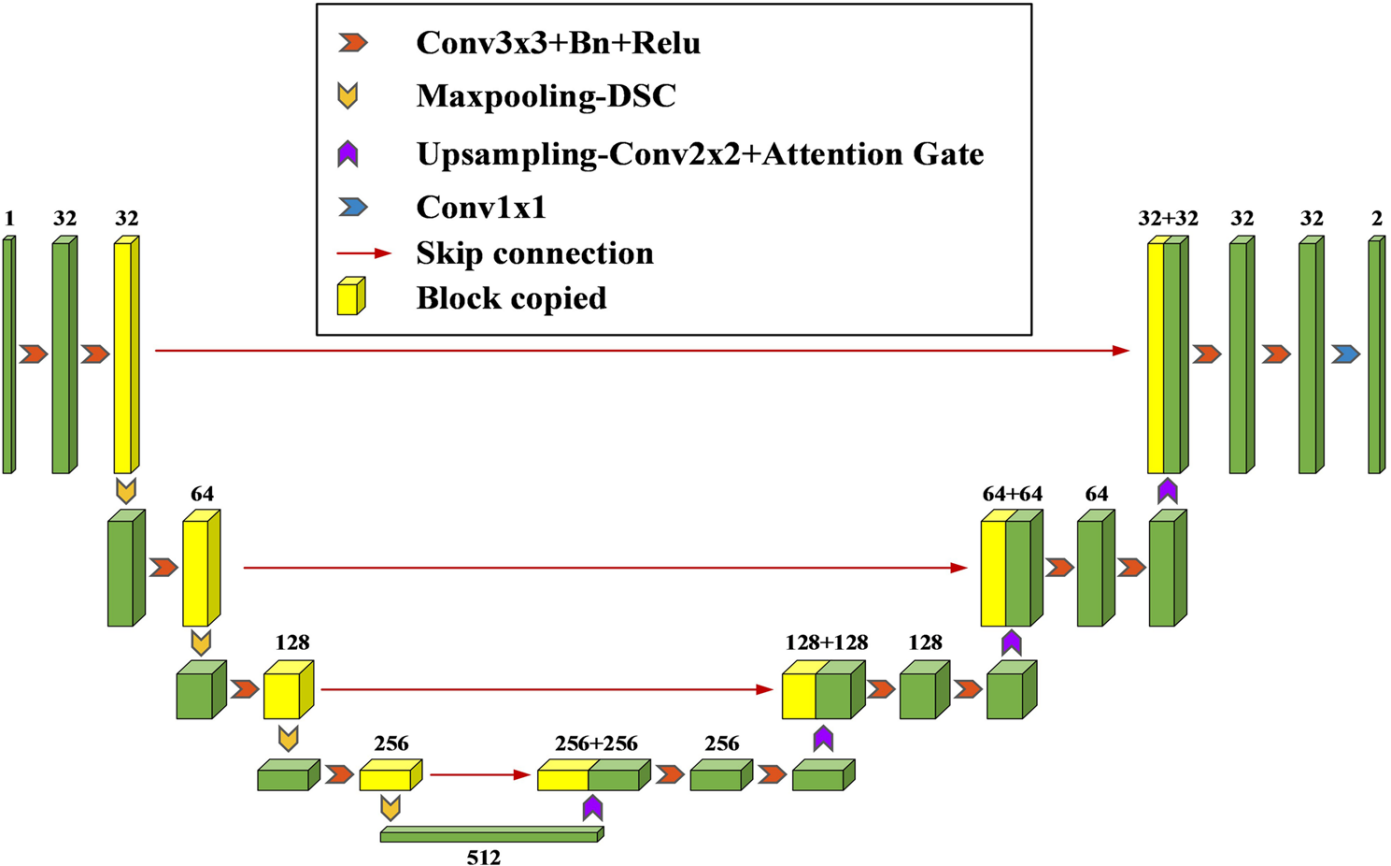
Structure of the 3D Attention U-Net with combined sampling

Incorporating convolution into the MaxPooling-DSC and UpSampling-Conv units boosts the model’s feature extraction capability and accelerates computational efficiency. It also addresses the lack of learnable parameters in traditional sampling layers, reducing feature loss during the sampling process and improving overall segmentation accuracy. The structure of the modified 3D Attention U-Net is shown in Fig. 4.

**Fig. 5:**
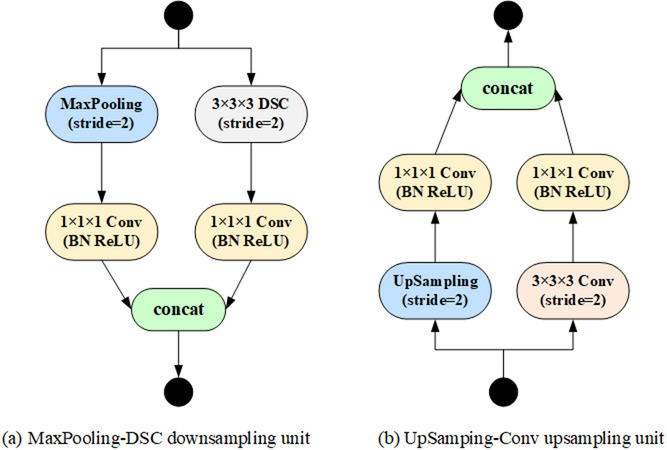
Improved sampling unit. **a **MaxPooling-DSC downsampling unit, (**b**) UpSampling-Conv upsampling unit

In the traditional 3D U-Net, the MaxPooling-DSC downsampling unit inherently includes a MaxPooling layer. This layer causes significant loss of semantic information (i.e., boundary segmentation accuracy) during feature map downscaling. For GBC images with diverse shapes and complex boundary features, we constructed a downsampling unit based on a position-constrained, combined-sampling 3D Attention U-Net (Fig. 5(a)). This unit enables parameter learning during the downsampling process. The feature map first passes through convolutional and MaxPooling layers. Depthwise separable convolution (DSC) is then applied to reduce the number of learnable parameters, which accelerates computation and mitigates overfitting. Next, a 1 × 1 × 1 convolution kernel processes the outputs from both layers to fuse multi-channel information and introduce non-linearity. Finally, the resulting feature maps are concatenated and input into the next convolutional layer to further enhance feature extraction. This process is expressed as:

1 $${F}_{1}^{{\prime\:}}=[\sigma\Big{(}{f}^{1\times\:1\times\:1}\big{(}MP({F}_{1})\big{)}\Big{)};\sigma\:({f}^{1\times\:1\times\:1}(DSC({F}_{1})))]$$

where $\:{F}_{1}$ and $\:{F}_{1}^{{\prime\:}}$ denote the input and output features, respectively; MP denotes the MaxPooling operation; DSC denotes the depthwise separable convolution layer; $\:{f}^{1\times\:1\times\:1}$ denotes the convolution operation with a 1 × 1 × 1 convolution kernel; $\:\sigma\:$ denotes the ReLU activation function; and “;” denotes the splicing of feature maps.

Similar to the MaxPooling-DSC downsampling unit, the upsampling structure of the UpSampling-Conv unit was modified (Fig. 5(b)). The feature map first passes through transposed convolutional and upsampling layers—transposed convolutions are deconvolution operations, while upsampling is the inverse of pooling. The resultant feature maps are processed with a 1 × 1 × 1 convolution kernel, concatenated, and input into the next convolutional layer. This process is expressed as:

2 $${F}_{2}^{{\prime\:}}=\Big{[}\sigma\Big{(}{f}^{1\times\:1\times\:1}\big{(}US({F}_{2})\big{)}\Big{)};\sigma\:\Big{(}{f}^{1\times\:1\times\:1}\big{(}TC({F}_{2})\big{)}\Big{)}\Big{]}$$

where $\:{F}_{2}$ and $\:{F}_{2}^{{\prime\:}}$enote the input and output features, respectively; $\:US$ denotes upsampling; and $\:TC$ denotes transposed convolution. The calculation process of $\:TC\left(F\right)$ is expressed as

3 $$\:TC{\left(F\right)}_{\left(i,j\right)}={\sum\:}_{\left(k,l,c\right)}^{\left(K,L,C\right)}{W}_{\left(k,l,c\right)}\bullet\:{y}_{\left(i+k,j+l,c\right)}$$

### Adaptive weight-based multimodal feature fusion

**Fig. 6:**
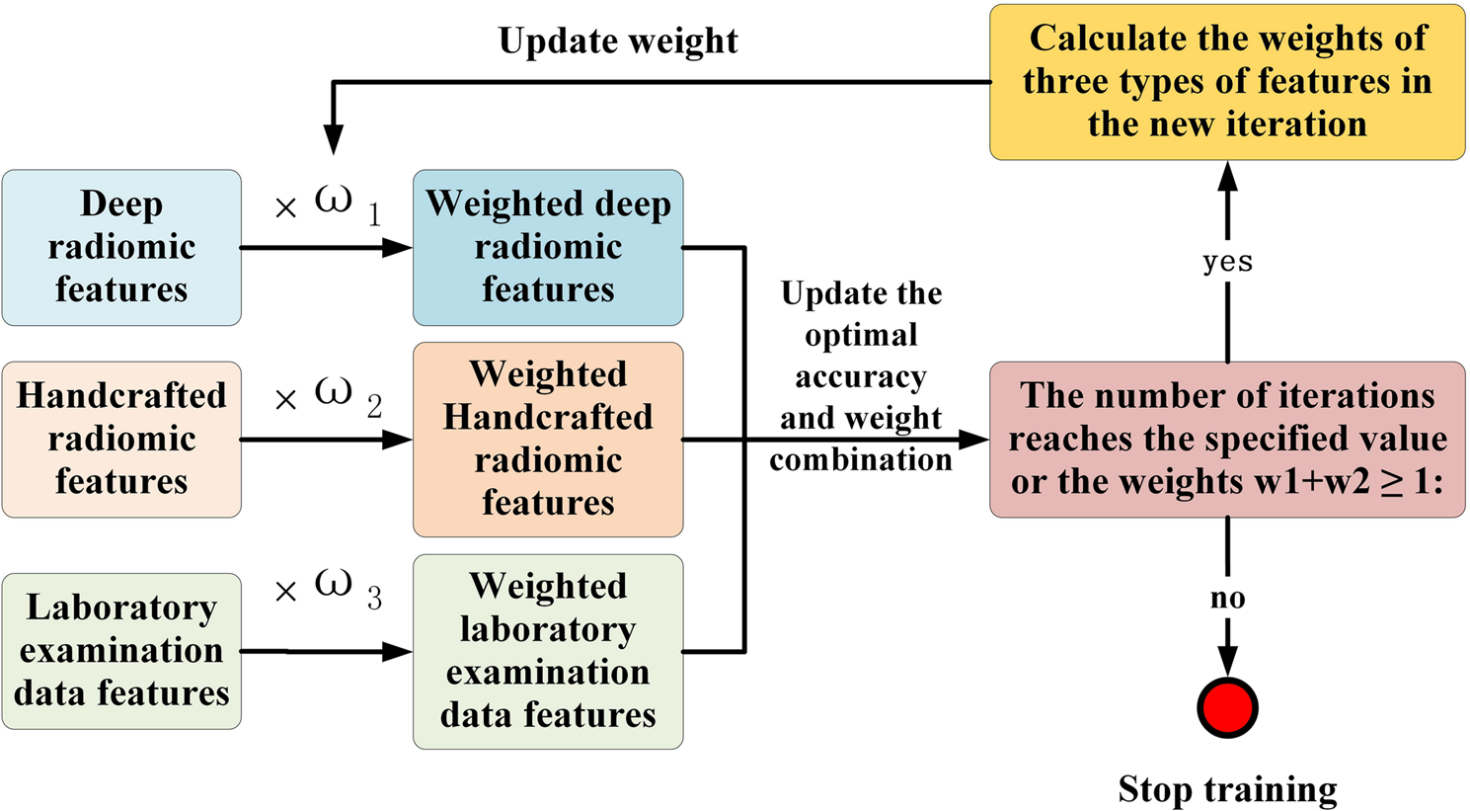
AWMFF weight update process

After extracting features from each data modality, we used an Adaptive Weight-Based Multimodal Feature Fusion (AWMFF) method to optimize feature integration. This method calculates feature weights based on the distribution and importance of modal features (e.g., variance, mean, Gaussian distribution). It also uses an improved Particle Swarm Optimization (PSO) algorithm to learn the optimal weight combination for maximizing diagnostic performance. For fusion, each modality’s features are multiplied by their respective weights. The weighted features are then linearly combined—the approach adopted in this study—to form the final fused feature representation. Other fusion methods (e.g., max pixel value) are also applicable and can be used based on specific task requirements. Post-processing steps can be added to the fusion pipeline as needed for individual applications. The AWMFF workflow is shown in Fig. 6.

In the AWMFF method, initial feature weights are determined by variance: features with a larger variance (more dispersed samples and higher representativeness) are assigned higher initial weights. This accelerates the algorithm’s convergence to the optimal weight solution. The diagnostic model first normalizes all multimodal features and assigns initial weights (w₁, w₂, w₃) with the constraints w₁ > w₂ > w₃ and w₁ + w₂ + w₃ = 1. The initial accuracy is obtained through the following steps: (1) weighting and fusing the normalized features; (2) concatenating the fused features in a fully connected layer; (3) inputting the concatenated features into a classifier for prediction. Weights are then updated using Eqs. (4)–(6) based on the current and best accuracy values. This update process is repeated until the maximum number of iterations is reached or the constraint w₁ + w₂ > 1 is violated.

4 $$\:{w}_{1}^{{\prime\:}}={w}_{1}+c\times\:random\left(\right)\times\:({acc}_{best}-{acc}_{now})$$

5 $$\:{w}_{2}^{{\prime\:}}={w}_{2}+c\times\:random\left(\right)\times\:({acc}_{best}-{acc}_{now})$$

6 $$\:{w}_{3}^{{\prime\:}}=1-{w}_{1}^{{\prime\:}}-{w}_{2}^{{\prime\:}}$$

where w1, w2, and w3 denote the modal feature weights from the previous iteration; w1’, w2’, and w3’ denote the updated weights of each modal feature; c denotes the learning factor (set to 0.5 in this study); the random () function generates a random floating-point number between − 1 and 1; $\:{acc}_{best}$ represents the best historical diagnostic accuracy; and $\:{acc}_{now}$ denotes the accuracy of the current iteration.

## Experiments

A total of 300 cases were randomly divided into a training-validation set (240 cases, 80%) and an independent test set (60 cases, 20%) using a stratified random sampling to ensure a balanced distribution of GBC and non-GBC cases in each subset. The 240 cases in the training-validation set were further split at a ratio of 8:2 into a training subset (192 cases, 80%) and a validation subset (48 cases, 20%), which were used for model training, hyperparameter optimization and early stopping to avoid overfitting. To ensure the robustness and reliability of model training, all models were trained using 5-fold cross-validation on the training-validation set. The independent test set (60 cases) was not involved in any model training or optimization process, and was only used to objectively evaluate the final generalization performance of the model.

### Experimental design

Laboratory examination data, handcrafted radiomic features, and deep radiomic features reflect disease characteristics at different dimensions and levels. To systematically explore the contribution of different feature combinations to model diagnostic performance and verify the superiority of multimodal feature fusion, we designed 8 groups of experiments with a core philosophy divided into three levels:

1. Single-feature model: Evaluate the diagnostic performance of a single feature type (laboratory examination data, handcrafted radiomic features, deep radiomic features) alone, and identify the optimal unimodal feature set for GBC diagnosis.

- Experiment 1: Diagnostic model based on feature-screened laboratory examination data.
- Experiment 2: Diagnostic model based on feature-screened handcrafted radiomic features.
- Experiment 3: Diagnostic model based on deep radiomic features.

2. Dual-feature model: Explore the synergistic effect of fusing any two feature types, and verify whether feature combination improves diagnostic performance compared with single feature models.

- Experiment 4: Diagnostic model based on feature-screened laboratory examination and handcrafted radiomic features.
- Experiment 5: Diagnostic model based on feature-screened laboratory examination and deep radiomic features.
- Experiment 6: Diagnostic model based on feature-screened handcrafted radiomic and deep radiomic features.

3. Three-feature model: Further evaluate the performance of fusing all three feature types, and compare the difference between simple unweighted concatenation (Experiment 7) and our proposed adaptive weighted fusion (Experiment 8). This is the core experimental step of the study, used to verify the effectiveness of the adaptive weight distribution methodology in multimodal feature fusion.

- Experiment 7: Diagnostic model based on deep radiomic, handcrafted radiomic, and laboratory examination features (unweighted concatenation).
- Experiment 8: Diagnostic model based on deep radiomic, handcrafted radiomic, and laboratory examination features with adaptive weighting.

**Fig. 7:**
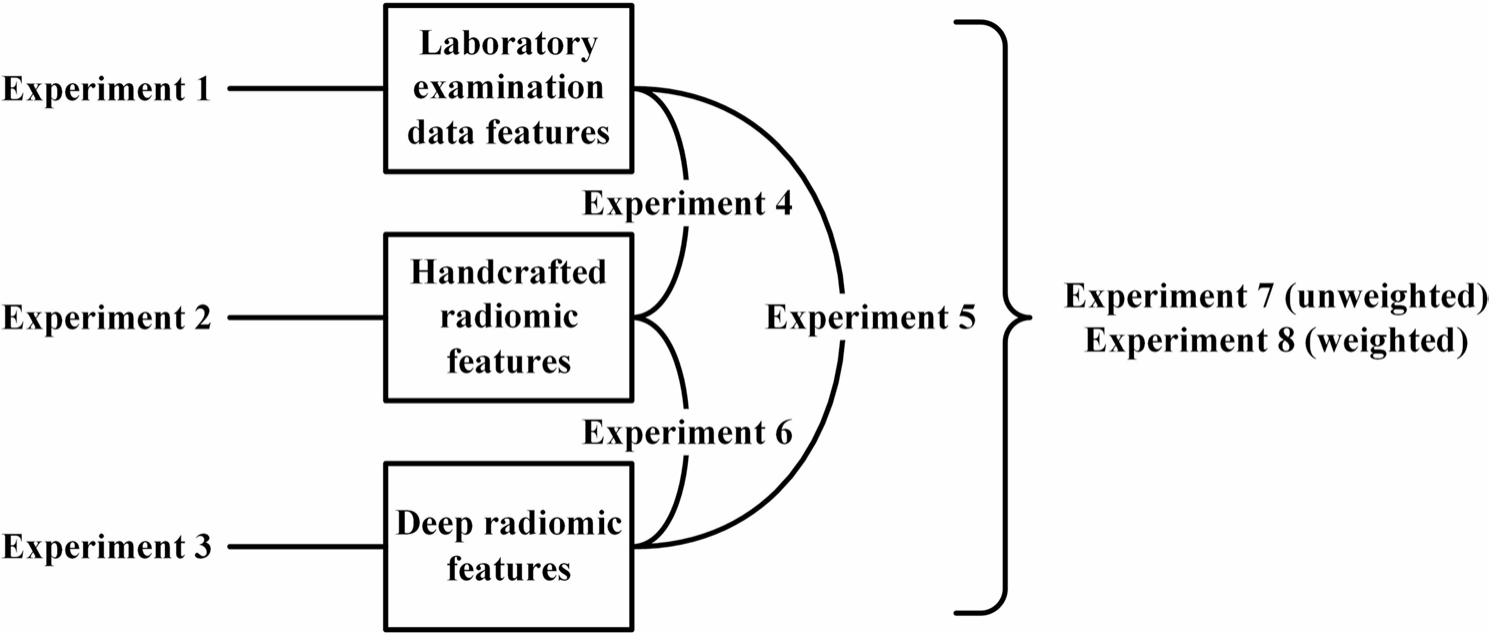
Grouping of experiments

The relationship between each experiment is shown in Fig. 7.

To ensure the scientific rationale, fairness, and reproducibility of all comparative experiments, the following principles were strictly followed: (1) Rationale: The eight experiments were designed to progressively evaluate the contribution of laboratory data, handcrafted radiomic features, and deep radiomic features in isolation, in pairs, and in full combination, to identify the optimal multimodal fusion strategy. (2) Fairness: All models were evaluated on the same independent test set (60 cases, 300 total cases) with identical train/validation/test split (80%/20%), same input data preprocessing, and same evaluation metrics (accuracy, sensitivity, specificity, F1-score, AUC). (3) Reproducibility: All experimental settings, including network structure, hyperparameters, optimization method, loss function, hardware, and software environment, were kept consistent.

### Evaluation metrics and experimental setup

The evaluation metrics for model prediction results include accuracy, specificity, sensitivity, precision, F1-score, and the receiver operating characteristic (ROC) curve. A larger area under the ROC curve (AUC) indicates better performance. The Dice coefficient is a widely used evaluation metric for image segmentation, which measures the similarity between predicted and gold standard labels. A Dice coefficient of 1 indicates perfect segmentation matching the gold standard, while a value of 0 indicates no overlap between the predicted and gold standard regions.

All experiments of GBC-DiagNet were conducted on a workstation with a configuration consistent with those commonly used in hospital research departments. The specific hardware and software parameters were as follows: Operating system: Linux Ubuntu 16.04; CPU: Intel Core i9-10940 × (3.30 GHz); GPU: NVIDIA TITAN RTX; Python versions: 3.7.0 and 3.10.0; CUDA: 11.0; CUDNN: 7.64; PyTorch: 1.7.1; TensorFlow: 1.14.0 and 2.11.0; and other relevant libraries (NumPy 1.19.2, Pandas 1.5.2). The hyperparameter settings of the model are as follows: the number of training epochs is 300, the batch size is 8, the Adam optimizer is adopted, the initial learning rate is 0.0001, the maximum learning rate is 0.005, and the dropout rate is set to 0.1.

## Results

### Comparison and analysis of the rough segmentation model

We tested five different segmentation models on the independent test set: 3D U-Net, 3D Attention U-Net, position-constrained 3D Attention U-Net, combined sampling-based 3D Attention U-Net, and 3D Attention U-Net with both position constraints and combined sampling. The segmentation results of each model are presented in Table 2.

**Table 2:** Comparison of segmentation results of each model on the test set

Model	Minimum Dice	Mean Dice (95% CI)	Maximum Dice
3D U-Net	0.374	0.641 (0.527–0.755)	0.940
3D Attention U-Net	0.333	0.736 (0.640–0.832)	0.941
3D Attention U-Net (position-constrained)	0.335	0.802 (0.714–0.890)	0.947
3D Attention U-Net (combined sampling)	0.378	0.774 (0.684–0.864)	0.940
3D Attention U-Net (position-constrained + combined sampling)	0.539	0.851 (0.790–0.912)	0.951

In comparison to the baseline 3D Attention U-Net, the addition of the combined sampling layer to its upsampling and downsampling workflows boosted the retention of both semantic information and learnable parameters. This enhancement improved the model’s segmentation accuracy for GBC boundaries and increased the overall Dice coefficient. Adding the position constraint network for post-processing of the preliminary segmentation results effectively reduced over-segmentation. The 3D Attention U-Net model with both position constraints and combined sampling outperformed all other models, including the original model that suffered from over-segmentation. Compared to the baseline 3D Attention U-Net (Mean Dice: 0.736), our proposed model achieved a Mean Dice of 0.851 (95% CI: 0.790–0.912), representing a 15.6% improvement. This quantitative gain in segmentation precision provides a more reliable ROI for subsequent feature extraction.

### Unimodal models vs. multimodal models

**Fig. 8:**
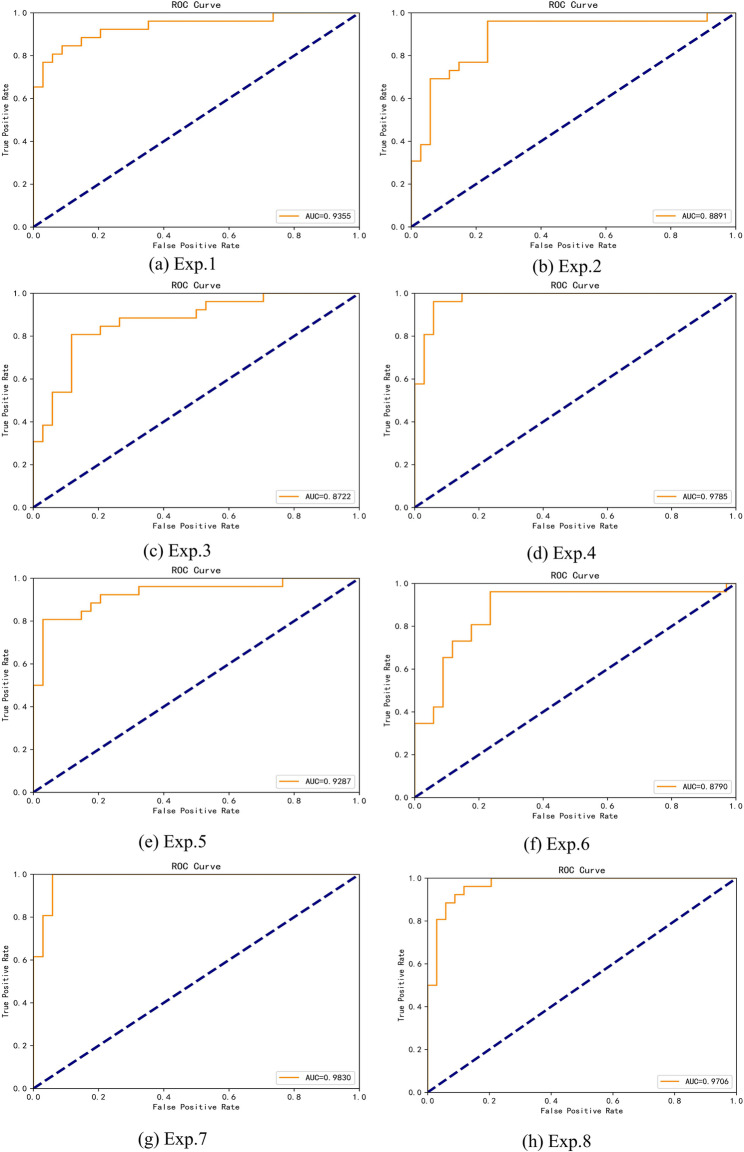
Comparison of ROC curves: (**a**) experiment 1, (**b**) experiment 2, (**c**) experiment 3, (**d**) experiment 4, (**e**) experiment 5, (**f**) experiment 6, (**g**) experiment 7, and (**h**) experiment 8

The accuracy, specificity, sensitivity, precision, F1-score, AUC, and corresponding 95% confidence intervals of experiments 1–8 are presented in Table 3. The corresponding ROC curves for Experiments 1–8 are shown in Figs. 8(a)–(h).

**Table 3:** Accuracy, specificity, sensitivity, precision, F_1_-score, and AUC of experiments 1–8

Experiment	Accuracy	Specificity	Sensitivity	Precision	F_1_-score	AUC
1	0.8000 ± 0.0144	0.8235 ± 0.0203	0.7692 ± 0.0350	0.7692 ± 0.0329	0.7692 ± 0.0022	0.9355 ± 0.0098
2	0.8167 ± 0.0145	0.8077 ± 0.0140	0.8235 ± 0.0227	0.7778 ± 0.0271	0.7924 ± 0.0278	0.8891 ± 0.0246
3	0.6000 ± 0.0044	**1.0000** **± 0.0000**	0.7690 ± 0.0012	**1.0000** **± 0.0000**	0.1428 ± 0.0013	0.8722 ± 0.0108
4	0.8833 ± 0.0190	0.8975 ± 0.0200	0.8462 ± 0.0219	0.8800 ± 0.0199	0.8627 ± 0.0081	0.9785 ± 0.0040
5	0.8833 ± 0.0151	0.8529 ± 0.0106	0.8077 ± 0.0171	0.8077 ± 0.0280	0.8077 ± 0.0260	0.9287 ± 0.0098
6	0.8000 ± 0.0165	0.7941 ± 0.0318	0.8077 ± 0.0192	0.7500 ± 0.0208	0.7778 ± 0.0062	0.8790 ± 0.0195
7	0.8833 ± 0.0185	0.8824 ± 0.0204	0.8846 ± 0.0104	0.8519 ± 0.0331	0.8679 ± 0.0143	**0.9830** **± 0.0049**
8	**0.9333** **± 0.0064**	0.9118 ± 0.0082	**0.9615** **± 0.0247**	0.8929 ± 0.0176	**0.9259** **± 0.0065**	0.9706 ± 0.0097

Note: Bold values indicate the highest performance achieved for each metric across all experiments, highlighted for readability purposes only

Compared with the unimodal models (Experiments 1–3), the dual-modal fusion models (Experiments 4–6) exhibited clear performance improvements across most evaluation metrics. This result indicates that integrating multiple feature types provides substantial diagnostic benefits over relying on any single modality alone. Further performance improvement was observed in Experiment 7, which used simple unweighted concatenation of all three modalities (deep radiomics, handcrafted radiomics, and laboratory data). This model outperformed all dual-modal approaches, confirming that fusing three feature levels captures more complementary diagnostic information than any pairwise feature combination. The best overall performance was achieved in Experiment 8, which used our proposed PSO-based adaptive weighting method for multimodal fusion. Compared with the simple unweighted concatenation in Experiment 7, Experiment 8 delivered higher accuracy, sensitivity, and F1-score. When the model reached its optimal accuracy, the learned adaptive weights were 0.3747 for deep radiomic features, 0.2039 for handcrafted radiomic features, and 0.4214 for laboratory data features. This adaptive mechanism automatically assigns greater importance to more discriminative modalities on a case-by-case basis, simulating how clinicians prioritize certain key clinical indicators during real-world GBC diagnosis.

Although Experiment 4 achieved a slightly higher AUC than Experiment 8, Experiment 8 markedly outperformed Experiment 4 in accuracy, sensitivity, and F1-score—metrics that better reflect practical clinical utility. At the same time, the AUC of Experiment 8 remained at a high level, indicating strong overall discriminative power for GBC diagnosis. Through Experiment 3 (deep radiomic features alone) demonstrated that deep learning features cannot fully capture tumor heterogeneity on their own. Only by integrating complementary feature types—especially with adaptive weighting—can the model achieve optimal performance across the majority of clinical evaluation metrics. On the same dataset, our weighted multimodal model improved accuracy by 14.28%, sensitivity by 16.76%, and F1-score by 16.85% compared with the best-performing unimodal model. These results demonstrate that our proposed approach effectively exploits richer, multi-dimensional diagnostic information that is unavailable to single-modality models.

In summary, while multimodal fusion itself provides clear advantages over unimodal and dual-modal strategies, the PSO-based adaptive weighting method is the key to unlocking the full potential of this three-modality complementary system. This method enables more robust and clinically relevant classification of GBC in clinical practice.

### Our model vs. state-of-the-art models

To compare the proposed model with state-of-the-art (SOTA) deep learning methods, we replaced the feature extractor in GBC-DiagNet (See Fig. 1) with the corresponding components from ResNet [25], DenseNet [26], MobileNet [27], ConvNeXt [28], and ViT [29]. To ensure fair and reproducible comparison, all compared state-of-the-art models (ResNet, DenseNet, MobileNet, ConvNeXt, ViT) were implemented and tested under the same dataset, preprocessing pipeline, training strategy, and evaluation protocol as the proposed GBC-DiagNet. The accuracy, specificity, sensitivity, precision, F1-score, and AUC of the models were computed, with the results presented in Table 4. General-purpose deep learning models are developed for natural image recognition, and their direct application in medical imaging faces significant domain-specific discrepancies. Our improved segmentation network is custom-designed for the GBC diagnosis task: by integrating the combined sampling layer and PCN, it effectively mitigates the over-segmentation issue and provides more accurate ROIs for subsequent feature extraction. Unlike other models that simply replace handcrafted features with deep learning features, GBC-DiagNet combines these two feature types to balance clinical interpretability and model performance. Additionally, our feature screening step further yields a key subset of highly discriminative features for GBC diagnosis. From the experimental results, it is evident that our model achieved an accuracy of 7.68%, an F1-score of 8.03%, and an AUC of 0.59% higher than the optimal comparison model (MobileNet).

**Table 4:** Accuracy, specificity, sensitivity, precision, F1-score, and AUC of other deep learning models

Model	Accuracy	Specificity	Sensitivity	Precision	F1-score	AUC
ResNet-50	0.8167 ± 0.0160	0.8529 ± 0.0287	0.7692 ± 0.0348	0.8000 ± 0.0249	0.7843 ± 0.0055	0.9253 ± 0.0144
ResNet-152	0.7333 ± 0.0100	0.9412 ± 0.0099	0.4615 ± 0.0165	0.8571 ± 0.0080	0.6000 ± 0.0235	0.8077 ± 0.0283
DenseNet-121	0.7167 ± 0.0050	0.9412 ± 0.0211	0.4231 ± 0.0363	0.8462 ± 0.0325	0.6000 ± 0.0218	0.9106 ± 0.0207
DenseNet-169	0.6833 ± 0.0083	**1.000** **± 0.0000**	0.3077 ± 0.0076	**1.0000** **± 0.0000**	0.4242 ± 0.0067	0.9140 ± 0.0041
MobileNet	0.8667 ± 0.0160	0.8235 ± 0.0254	0.9231 ± 0.0190	0.8000 ± 0.0218	0.8571 ± 0.0229	0.9129 ± 0.0160
ConvNeXt_T	0.7000 ± 0.0161	0.5000 ± 0.0393	0.9615 ± 0.0027	0.5952 ± 0.0111	0.7353 ± 0.0131	0.9321 ± 0.0056
ConvNeXt_S	0.7167 ± 0.0134	0.5000 ± 0.0236	**1.0000** **± 0.0000**	0.6047 ± 0.0106	0.7536 ± 0.0140	0.9140 ± 0.0148
ViT-B/16	0.8000 ± 0.0076	0.9118 ± 0.0035	0.6538 ± 0.0350	0.8500 ± 0.0261	0.7391 ± 0.0118	0.8982 ± 0.0134
ViT-B/32	0.7500 ± 0.0162	0.5588 ± 0.0080	1.0000 ± 0.0000	0.6341 ± 0.0321	0.7761 ± 0.0125	0.9649 ± 0.0019
ViT-L/16	0.8333 ± 0.0072	0.7353 ± 0.0068	0.9615 ± 0.0192	0.7353 ± 0.0266	0.8333 ± 0.0154	0.8971 ± 0.0102
ViT-L/32	0.7833 ± 0.0142	0.6471 ± 0.0325	0.9615 ± 0.0110	0.6757 ± 0.0028	0.7937 ± 0.0170	0.8213 ± 0.0189
**Ours**	**0.9333** **± 0.0064**	0.9118 ± 0.0082	0.9615 ± 0.0247	0.8929 ± 0.0176	**0.9259** **± 0.0065**	**0.9706** **± 0.0097**

Note: Bold values indicate the highest performance achieved for each metric across all compared models, highlighted for readability purposes only

### Sample size power analysis

To validate the adequacy of the study sample size and ensure the statistical rigor of the reported performance gains (7.68% accuracy improvement over SOTA models), a sample size power analysis was conducted based on the binary classification task (GBC vs. non-GBC). The effect size was quantified using Cohen’s h coefficient to reflect the accuracy difference between the two groups: the accuracy of the proposed model was 93.33% (Exp.8 result), and the accuracy of the optimal SOTA model (MobileNet) was 86.67% (Table 4). This yielded a Cohen’s h coefficient of 0.182, which represents a moderate effect size—consistent with the reasonable range of performance improvements for clinical AI models. The significance level (α) was set to 0.05 (two-tailed test), in line with common standards in the medical and AI research fields. The target statistical power (1-β) was set to ≥ 80%, ensuring that the probability of detecting a true performance difference between the models is no less than 80%. A paired study design was adopted where the same 60-case independent test set was used to evaluate both our proposed model and all SOTA models. The total cohort size was 300 cases (240 cases for training and validation, 60 cases for testing), sourced from two Grade A tertiary hospitals in Shanghai. G*Power 3.1 software was employed for the power analysis of paired binary proportion tests.

The power analysis results showed that with a total sample size of 300, a Cohen’s h of 0.182, and an α of 0.05 (two-tailed), the actual statistical power of this study was 89.7%, This value exceeds the preset threshold of 80%. Reverse validation indicated that for a target power of 80%, an α of 0.05, and a Cohen’s h of 0.182, the minimum required sample size was 228 cases. The actual sample size of this study (300 cases) was significantly larger than this minimum requirement, confirming that the cohort size is sufficient to reliably detect the reported 7.68% accuracy improvement over SOTA models.

## Discussion

GBC diagnosis remains a major clinical challenge worldwide. To emulate clinicians’ diagnostic approach—typically relying on diverse clinical data sources—we proposed a two-stage GBC diagnostic model integrating heterogeneous multimodal data, which outperformed both unimodal models and state-of-the-art (SOTA) deep learning methods.

### Comparison with previous studies

Multimodal fusion methods have demonstrated high accuracy in diagnosing various diseases, and many researchers have applied these techniques to clinical diagnosis [30, 31]. For GBC diagnosis, however, most existing models rely on unimodal data, such as laboratory examination results or CT images [32, 33]. For example, Kinugasa et al. [34] developed a model based solely on laboratory examination data to generate cancer probability scores. Kumar et al. [35] combined thiobarbituric acid reactive substances, chemiluminescent microparticle immunoassay data, and tumor marker features to assist clinical decision-making. Zhang et al. [36] used chi-square tests to assess feature dependencies and input the selected features into a random forest algorithm, which provided valuable diagnostic support. Basu et al. [37] proposed a CNN-based GBC detection network that partially addresses the challenges of low image quality and noise in ultrasonography. Relatively few studies have explored multimodal data fusion for GBC diagnosis [38]. Unlike these prior studies, the proposed model integrates heterogeneous multimodal data to significantly enhance the performance of deep learning-based GBC diagnosis.

Our model leverages multimodal data (including imaging and laboratory data) for GBC diagnosis, so we compared it with SOTA GBC diagnostic models that use imaging data (Table 5). In addition to contrast-enhanced CT,, these studies used ultrasound and ^18^F-FDG PET as imaging modalities. The patient datasets in three of the studies did not exceed 300 cases, which aligns with the low global incidence of GBC. Three of the studies employed deep learning-related methods [32, 33, 37]. Compared with two studies of similar data size [33, 37], our model achieved greater AUC and accuracy, highlighting the value of multimodal data integration in this research. Our model also outperformed the only other multimodal study [38] in terms of AUC, further supporting the value of the proposed multimodal approach. Notably, Fujita et al. [32] reported slightly higher accuracy and AUC than our model, but their study included only 49 patients—far fewer than our 300-case cohort. The small sample size in their study may limit the generalizability of their results, whereas our larger, multicenter dataset enhances the robustness and clinical applicability of our findings.

**Table 5:** Comparison with SOTA image-based GBC diagnostic models

Model	Modality	Dataset Size	Method	Spec.(%)	Sens.(%)	Acc.(%)	AUC
Fujita[32]	Contrast-enhanced CT	49	ResNet50	98.0	98.8	98.5	0.998
Basu [37]	Ultrasound	218	GBCNet	95.0	97.6	91.0	0.970
Xiang[33]	Contrast-enhanced CT	278	ResNet50	76.0	85.0	80.0	0.857
Li [38]	^18^F-FDG PET, patient signs, medical history and laboratory examination	122	Student’s t test, the Mann‒Whitney test and the chi-squared test	73.5	90.9	/	0.899
Ours	Contrast-enhanced CT, laboratory examination	300	GBC-DiagNet	91.2	96.2	93.3	0.971

### Limitations and future work

**Fig. 9:**
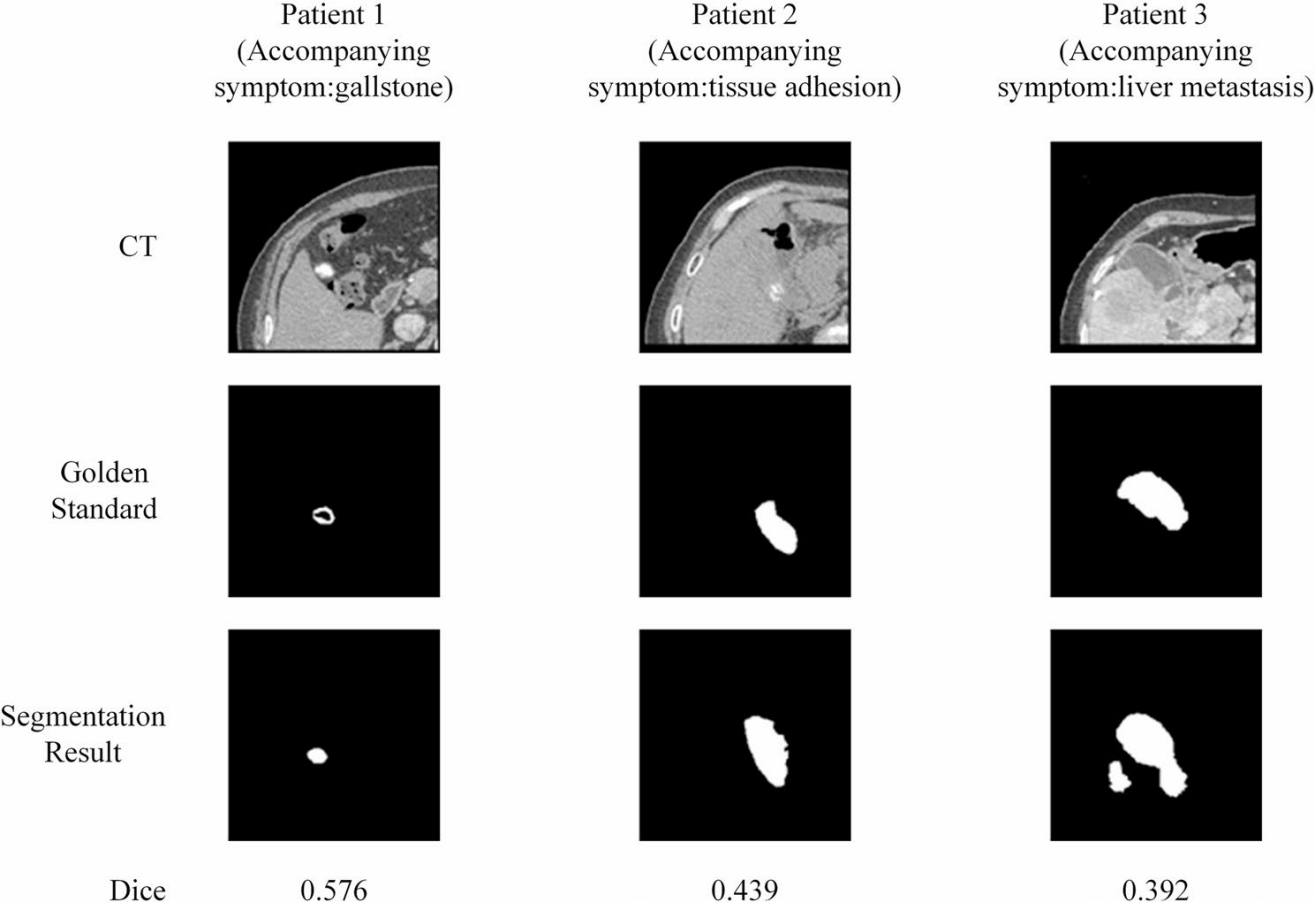
Example segmentation results for patients with suboptimal segmentation performance

Despite the promising results, this study has several limitations. As illustrated in Fig. 9, the image segmentation results from the first stage of our model were not consistently satisfactory in all cases. The suboptimal segmentation in some cases is mainly attributed to the frequent coexistence of GBC with other malignant or pathological conditions, such as liver metastases, lymph node metastases, tissue adhesions, gallstones, and extreme anatomical distortion (e.g., severe post-surgical adhesions, end-stage liver disease, or other conditions that disrupt normal gallbladder and peri-gallbladder anatomy). These concurrent lesions or comorbidities significantly alter the imaging appearance of the gallbladder and surrounding structures, reducing the discriminative power of contrast-enhanced CT features for accurate segmentation. In affected patients, poor segmentation results are mainly driven by the presence of these adjacent or concurrent lesions. Unfortunately, the limited number of training cases containing these specific types of concurrent lesions makes it difficult for the model to adequately learn the distinctive imaging characteristics of such complex scenarios, resulting in less-than-ideal segmentation performance in this subgroup.

Notably, three cases with adequate image quality and consistent annotations still yielded low Dice coefficients. Analysis of the model’s Attention maps revealed that the model overly focused on high-intensity regions of adjacent metastatic lesions rather than the gallbladder boundaries. This reflects a key limitation of the current Attention mechanism: it prioritizes high-contrast regions but fails to distinguish the gallbladder from adjacent lesions with similar enhancement features. To address this issue, we plan to add a lesion-aware attention branch to the 3D Attention U-Net, integrating clinical prior knowledge (e.g., the typical anatomical position of gallbladder lesions relative to the liver) to better differentiate the gallbladder from surrounding abnormal tissues.

In summary, to address the identified limitations and enhance model performance, our future research will focus on three key directions: (1) Expanding the training dataset to include more cases with concurrent lesions, enabling the model to learn more diverse anatomical and imaging features of GBC; (2) Integrating clinical prior knowledge into the segmentation network to strengthen the model’s ability to discriminate the gallbladder from adjacent pathological tissues; (3) Adopting a multi-task learning framework that jointly optimizes lesion segmentation and classification tasks, allowing the model to leverage diagnostic information to guide more precise regional delineation of the gallbladder and its lesions.

### How to use GBC-DiagNet in clinical practice

GBC-DiagNet is specifically designed as a second-opinion auxiliary tool to support rather than replace clinical expertise. Its workflow emphasizes safe, transparent integration into routine clinical decision-making. Each model output includes a confidence score (0–100%) and a segmentation quality assessment (Dice score). For high-confidence predictions (≥ 85%) with reliable segmentation (Dice ≥ 0.7), the result can serve as a confirmatory reference to support clinician decisions. Low-confidence cases (< 60%) or those with poor segmentation (Dice < 0.6, e.g., due to comorbidities) are automatically flagged as high-uncertainty, with explanations highlighting potential causes to alert clinicians to exercise additional caution.

In cases of discordance between AI suggestions and clinician judgment, a structured reconciliation protocol is recommended: (1) Review of interpretability outputs (feature importance, fusion weights, segmentation masks) to pinpoint the source of discrepancies; (2) Optional manual ROI adjustment or addition of clinical context for model re-analysis; and (3) Escalation to a multidisciplinary team (radiologists, hepatobiliary surgeons, pathologists) if discrepancies persist, incorporating both AI reasoning and clinician notes to reach a consensus. All outputs—including inputs, weights, and scores—are logged in the electronic medical record (EMR) as an “AI auxiliary opinion” for traceability, while ultimate diagnostic responsibility remains with the clinician. This positions GBC-DiagNet as a practical “safety net” to minimize missed diagnoses and reduce overtreatment in ambiguous gallbladder lesions.

## Conclusions

GBC is a highly malignant gastrointestinal tumor, and early, accurate diagnosis is critical for improving patient survival. In this study, a two-stage multimodal fusion diagnostic model named GBC-DiagNet was proposed, which integrates contrast-enhanced CT images and laboratory examination data. On the independent test set, our model achieved an accuracy of 93.3%, a sensitivity of 96.2%, a specificity of 91.2%, and an AUC of 0.9706. Compared with traditional unimodal deep learning networks and existing GBC diagnostic models, GBC-DiagNet achieved more favorable values across key diagnostic metrics. These quantitative findings confirm that the proposed multimodal fusion strategy effectively improves the accuracy and reliability of GBC auxiliary diagnosis. Furthermore, the model provides interpretable and clinically meaningful decision support for clinicians, thus facilitating the preoperative non-invasive diagnosis of GBC in clinical practice.

## Data Availability

The complete code and dataset are presented in the online repository: https://github.com/yinziming/Artificial-Intelligence-of-Medical-Group/tree/main/2133327481-gaohongyu/multimodal-model-for-gallbladder-cancer-main.

## Abbreviations

GBC: Gallbladder cancer
CT: Computed tomography
CI: Confidence interval
ROI: Regions of interest
FC: Fully connected layer
PCN: Position-constraint network
ROC: Receiver operating characteristic
AUC: Area under the curve
Spec: Specificity
Sens: Sensitivity
Acc: Accuracy
CNN: Convolutional neural network
EMR: Electronic medical record
PSO: Particle swarm optimization
DSC: Depthwise separable convolution
